# Enhertu (Fam-trastuzumab-deruxtecan-nxki) – Revolutionizing treatment paradigm for HER2-Low breast cancer

**DOI:** 10.1016/j.amsu.2022.104665

**Published:** 2022-09-16

**Authors:** Tasmiyah Siddiqui, Payal Rani, Tayyaba Ashraf, Aayat Ellahi

**Affiliations:** a Department of Internal Medicine, Dow University of Health Sciences, Karachi, Pakistan; b Department of Internal Medicine, Jinnah Sindh Medical University, Karachi, Pakistan

**Keywords:** HER2-Low breast cancer, Enhertu (trastuzumab deruxtecan), Destiny breast 04 trial, Interstitial lung disease, FDA-Approved

Breast cancer is the second-leading cause of cancer-related death and the most prevalent malignancy in the globe [1]. Lumpectomy, mastectomy, radiotherapy, chemotherapy, immune cell therapy, endocrine therapy, and targeted therapy have been used as treatments for breast cancer patients depending upon the type, size, stage, grade, and sensitivity of cancer cells. But recently, revolutionizing the treatment paradigm, unresectable or metastatic HER2-low breast cancer that has previously been treated with chemotherapy can now be treated with Enhertu (fam-trastuzumab-deruxtecan-nxki), the first targeted therapy approved by U.S Food and Drug Administration (FDA) [2].

HER2-positive breast cancers are those that meet a certain high threshold of Human Epidermal Growth Factor Receptor (HER) Molecules 3+ Immunohistochemical (IHC) score. Conversely, breast cancers with HER2 levels below the threshold are categorized as HER2-negative breast cancers (IHC score 0 or 1+). However, in between the HER2-positive and HER2-negative subtypes of breast cancer, there is another subtype of breast cancer called HER2-low that expresses trace levels of HER2 proteins with IHC scores of +1 or +2. The FDA estimates that in 2022, U.S will witness 287,850 novel cases of female breast cancer. Approximately 80–85% of the recent cases were formerly classified as HER2-negative, with 60% of patients now classified as HER2-Low [2]. Although available targeted therapies for HER2-positive breast cancers have been disappointing in HER2-low breast cancers, Trastuzumab deruxtecan (T-DXd) has demonstrated extremely promising results and can now be utilized maximally not only to treat HER2-positive breast cancers but also this newly discovered HER2-low breast cancer, which could only receive endocrine therapy or chemotherapy prior to Enhertu's approval by the FDA.

Fam-trastuzumab deruxtecan-nxki, a targeted therapy is antibody drug conjugate (ADC) which is directed for HER2 cancers. It has two parts, humanized anti-HER-2 monoclonal antibody and topoisomerase inhibitor payload “deruxtecan” which are covalently attached through a tetra peptide-based cleavable linker. Once bound to the HER-2 receptor on tumor cells, cell internalizes the antibody along with the bounded deruxtecan and linker cleavage occurs through lysosomal enzymes which release deruxtecan. After its release, it impairs the replicating ability of cell at the time of cell division, causing DNA damage and leading to apoptotic cell death [3].

This drug has shown a promising objective response rate in a variety of cancers, namely non-small cell lung cancer (NSCLC) and colorectal cancer that express HER2 protein [4]. Likewise, it has been a part of major phase 1 and phase 2 clinical trials which displayed the safety and efficacy of this drug for cancers including HER2-positive breast cancer that are unable to be removed through surgery or has metastasized to other organs of the body following anti-HER2 breast cancer treatment either in a metastatic setting or recurrence of disease throughout or in the course of six months of completion of therapy; and HER2+ advanced stomach cancer [[5], [6], [7]] According to DESTINY-BREAST01 Phase 2 trials, the confirmed response rate of 60.9% was seen in the patients who received the dose. The 6 month overall survival was estimated to be 93.9% and 86.2% at 12 months [8]. Recently, it has been found to have promising effects in the DESTINY-BreastO4 phase 3 trial in which 555 adult patients were matriculated with inoperable or metastatic HER2-Low Breast Cancer and the results revealed significantly longer progression-free survival (PFS) of 9.9 months and overall survival of 23.4 months [9]. These drastic improvements in the rate of survival and remission led to the accelerated approval of this drug by the FDA.

However, it is not devoid of side effects and requires attentive surveillance. The established adverse reactions seen in patients were nausea, reduced appetite, vomiting, musculoskeletal pain, fatigue, diarrhea, alopecia, and constipation. Approximately 28% of patients had adverse reactions, and 16% of people had to stop receiving the drug permanently during the trial. Other adverse events of special interest include pneumonitis and sepsis, with 12% of patients suffering from and 3 dying of Interstitial Lung Disease (ILD) [9]. These adverse events are the principal cause of the necessary boxed warning that forewarns health care professionals of ILD and embryo foetal toxicity associated with it.

Although this drug is yet to be tested in larger trials and comes with potential adverse events, the PFS of about 10 months it shows is its significant advantage over other drugs with a PFS of 5 months [9]. Additionally, its overall survival difference of 23.4 months compared with 16 months of other drugs is yet another remarkable and clinically meaningful finding [9]. Since it specifically targets HER2-expressing cancer cells, its use may extend into subsets of stomach, lung, colorectal, and other cancers that express HER2 as well. While this drug does not eradicate the cancer completely and cannot be called a cure, given its robust efficacy and ground-breaking effects, it may prove to be an efficacious alternative to standard chemotherapy, which targets not only cancer cells but healthy cells as well.

## Ethical approval

N/A.

## Sources of funding

None.

## Author contribution

Tasmiyah Siddiqui proposed the idea.

Tasmiyah Siddiqui, Payal Rani and Tayyaba collected the data and completed the manuscript.

Aayat Ellahi finalized the manuscript did the proof-reading and submission of article. All authors approved the final version of article.

## Registration of research studies

1. Name of the registry: N/A.

2. Unique Identifying number or registration ID: N/A.

3. Hyperlink to your specific registration (must be publicly accessible and will be checked): N/A.

## Guarantor

Tasmiyah Siddiqui.

Payal Rani.

Tayabba Ashraf.

Aayat Ellahi.

## Consent

N/A.

## Declaration of competing interest

None.
